# One-year results of half-dose versus one-third-dose photodynamic therapy in chronic or recurrent central serous chorioretinopathy

**DOI:** 10.1186/s12886-020-01796-0

**Published:** 2021-01-12

**Authors:** Jirarattanasopa Pichai, Banchasakjaroen Vanchalerm, Ratanasukon Mansing

**Affiliations:** Department of Ophthalmology, Faculty of Medicine, Prince of Songkla University, Songkhla, 90110 Thailand

**Keywords:** Photodynamic therapy (PDT), Half-dose PDT, One-third-dose PDT, Central serous chorioretinopathy (CSC)

## Abstract

**Background:**

Central serous chorioretinopathy (CSC) is characterized by an accumulation of subretinal fluid (SRF) in the macula. It is usually treated by laser photocoagulation or photodynamic therapy (PDT) with consisting of different doses and power. This study aimed to compare the efficacy of half-dose PDT and one-third-dose PDT in chronic or recurrent CSC.

**Methods:**

A retrospective review of patients with chronic or recurrent CSC who were treated with either a half-dose or one-third-dose PDT, and had follow up 12 months afterwards. Best-corrected visual acuity (BCVA), central retinal thickness (CRT) and resolution of subretinal fluid (SRF) at baseline as well as 1, 3, 6 and 12 months post-PDT were assessed.

**Results:**

Forty-six eyes and 20 eyes received half-dose and one-third-dose PDT, respectively. The study showed efficacy of the one-third-dose PDT compared with half-dose PDT in BCVA improvement (0.10±0.04 logMAR for one-third-dose versus 0.17±0.04, for half-dose, *P*=0.148) and CRT improvement (125.6±24.6 μm for one-third-dose versus 139.1±16.54, for half-dose, *P*=0.933) at 12 months. The SRF recurrence rate was significantly higher in the one-third-dose PDT group compared with the half-dose PDT group (40.0% versus 15.2%, *P*=0.027) at 12-months.

**Conclusion:**

At 12 months, the one-third-dose PDT was effective in terms of BCVA and CRT improvement, when compared with half-dose PDT. However, this study showed that one-third-dose PDT had a higher recurrence rate of SRF.

## A brief summary statement

At 12 months, the one-third-dose PDT was effective in term of BCVA and CRT improvement, when compared with half-dose PDT. However, this study showed that the one-third-dose PDT had a higher recurrence rate of SRF.

## Background

Central serous chorioretinopathy (CSC) is characterized by an accumulation of subretinal fluid (SRF) in the macula; with or without serous detachment of the retinal pigment epithelium (RPE), caused by choroidal vascular hyperpermeability [1, 2] Acute CSC spontaneously resolves within 3–4 months in most patients, but patients who have persistent or recurrent SRF require treatment [1–4].

Laser photocoagulation is generally used to treat extrafoveal leakage. However, chronic CSC, with broad or indistinct leakage, and CSC with subfoveal or juxtafoveal leakage are difficult to treat with laser photocoagulation because of difficulty in localizing the leakage point. Additionally, there is the possibility of serious complications, such as RPE atrophy, permanent scotoma and secondary choroidal neovascularization (CNV) [4–7]. Photodynamic therapy (PDT) with verteporfin has been reported to be effective for reducing SRF; thereby improving the visual acuity in these patients [8–13]. However, post-PDT complications, such as RPE change, choroidal ischemia and secondary CNV [14–16], have motivated studies of the effect of lowering the dose of verteporfin, or decreasing the laser power energy (decreasing the fluence), so as to minimize post-PDT complications. Accordingly, many studies have reported the efficacy of half-dose PDT and half-fluence PDT in treatment of acute and chronic CSC, without serious complications [17–23].

Because of the variation in verteporfin doses being used for CSC, Zhao et al. [24, 25] performed a study showed that 30% verteporfin seemed to be safe and effective in the treatment of acute CSC. Due to issues with reimbursement, some patients in this study, conducted the Prince of Songkla University (PSU) hospital, were treated with one-third-dose PDT rather than half-dose PDT.

The purpose of this present study was to retrospectively compare the 1-year results for visual acuities, central retinal thickness (CRT), SRF and recurrence rate between half-dose PDT and one-third-dose PDT in patients with chronic or recurrent CSC.

## Methods

We retrospectively reviewed medical records and images from patients who received half-dose PDT or one-third-dose PDT for chronic or recurrent CSC between January, 2012 and December, 2017; at the Department of Ophthalmology, Songklanagarind Hospital, PSU, Songkhla Province, Thailand. Our study was approved by the Institutional Review Board of Songklanagarind Hospital, PSU, and adhered to the guidelines of the Declaration of Helsinki.

We classified CSC into two types; chronic CSC; defined as based on symptoms persisting for longer than 3 months, and recurrent CSC; defined as based on new symptoms in the same eye of a patient with visual deficits from an earlier episode of CSC.

The inclusion criteria were as follows: (1) aged between 20 and 70 years; (2) chronic or recurrent CSC that was treated with half-dose PDT, or one-third-dose PDT; (3) presence of SRF involving the macula, as evident on spectral-domain optical coherence tomography (SD-OCT); and (4) the presence of active leakage on fluorescein angiography (FA).

The exclusion criteria were as follows: (1) previous PDT or intravitreal injections of anti-vascular endothelial growth factors (VEGF) or steroids; (2) previous intraocular surgery; (3) other macular abnormalities; such as CNV, polypoidal choroidal vasculopathy (PCV) or other maculopathy; and (4) follow-up of less than 12 months, or missing data, including as best-corrected visual acuity (BCVA), OCT images at baseline, or 1, 3, 6 or 12 months after treatment.

The data collected included: patient gender, age, weight, height, laterality of visual impairment, duration of symptoms, type of CSC, leakage type of CSC on FA, spot size of PDT treatment, BCVA and CRT at baseline and at 1, 3, 6 and 12 months after treatment. The data were recorded from electronic medical records via the hospital information system, at the Department of Ophthalmology, Faculty of Medicine, PSU, Songkhla Province, Thailand.

The main outcome measures were the improvement in BCVA and CRT, and the presence of SRF at the 1-, 3-, 6- and 12-month follow-ups after PDT treatment. Evaluation of macular detachment and CRT were performed using an SD-OCT machine [(Spectralis®, Heidelberg Engineering, Heidelberg, Germany) or (Cirrus OCT®, Carl Zeiss Meditec, Inc., Dublin, CA)]. We used the same machine for each patient during follow-ups. All patients underwent simultaneous FA and indocyanine green angiography (ICGA) [Heidelberg retinal angiography, Heidelberg Engineering, Heidelberg, Germany] at baseline, for confirmation of the diagnosis and planning of PDT treatment.

PDT was performed by administering a half-dose (3 mg/m^2^), or one-third-dose (2 mg/m^2^) of verteporfin (Visudyne®, Novartis AG, Basel, Switzerland). All patients were randomized, by issue with reimbursement. Verteporfin was infused for 8 min. The laser (wavelength 689 nm, with a total light energy of 50 J/cm^2^ for 83 s), was delivered to the area of angiographic leakage observed on FA; with an associated area of choroidal hyperpermeability on ICGA at 10 min after infusion. After treatment, all patients were instructed to avoid sunlight for 48 h. Informed consent for PDT was obtained from each patient, after having a discussion of the potential risks and benefits.

Statistical analysis was performed using SPSS (version 11.0, SPSS Inc., Chicago, IL). Linear fixed and random-effects models were used to compare BCVA and CRT between the two dosage groups, including the changes in BCVA and CRT within each group. The cumulative numbers of eyes with complete subretinal fluid reabsorption and recurrence of subretinal fluid were tested using Fisher exact test. *P*-values of < 0.05 were considered statistically significant.

## Results

**Fig. 1:**
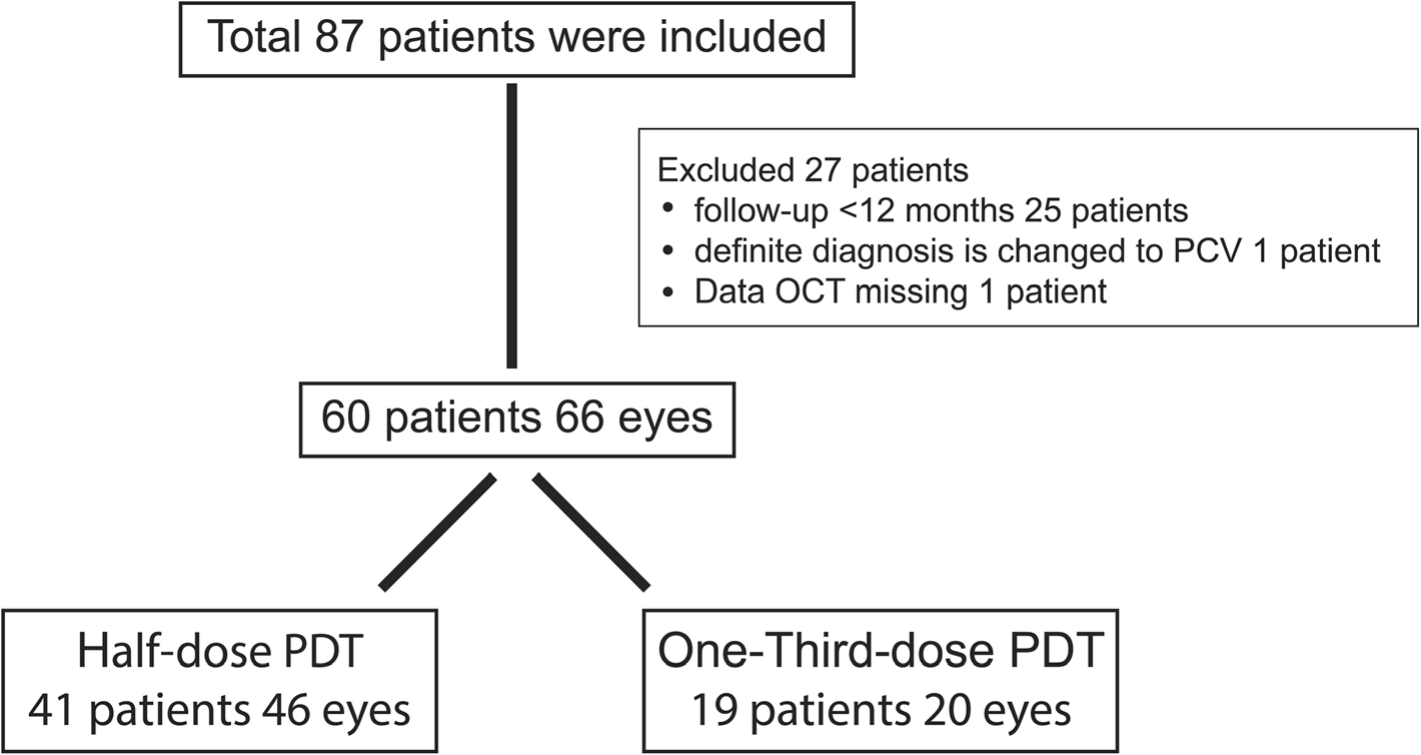
Flowchart of patients and eyes included in, and excluded, from the study. Abbreviations: OCT, optical coherence tomography: PCV, polypoidal choroidal vasculopathy: PDT, photodynamic theraphy

A total of 87 patients with chronic or recurrent CSC were included. Twenty-seven patients were excluded because: their follow-up was less than 12 months (25 patients), the diagnosis was changed to PCV (1 patient) or their follow-up OCT data were missing (1 patient). Finally, data from 60 patients (66 eyes) were analyzed. From this, 41 patients (46 eyes) had received half-dose PDT and 19 patients (20 eyes) had received one-third-dose PDT (Fig. 1).

The baseline demographic data and clinical characteristics of the patients in the two treatment groups are summarized in Table 1. There were no statistically significant differences in gender, laterality, body mass index, duration of symptoms, type of CSC, leakage type on FA, spot size of PDT, initial BCVA or CRT. However, the half-dose PDT group was older than the one-third-dose PDT group (*P*=0.03).

**Table 1:** Comparison of demographic data for the two treatment groups

Characteristic	Half-dose PDT group (*n* = 46 eyes/41 patients)	One-third-dose PDT group (*n* = 20 eyes/19 patients)	*P*-value
Age, mean ± SD (years)	51.51 ±7.8	46.47±8.88	0.030*
Sex, No. (%)			
-Male	29 (70.7)	15 (78.9)	0.754
-Female	12 (29.3)	4 (21.1)	
Study eye, No. (%)			
-Right	25 (54.3)	10 (50.0)	0.793
-Left	21 (45.7)	10 (50.0)	
BMI, mean ±SD, kg/m^2^	24.07±3.80	24.06 ±3.18	0.994
Duration of symptoms, mean ±SD, (months)	9.43 ±8.84	12.35 ±8.13	0.212
Group types, No. (%)			
-Chronic	39 (84.8)	13 (65.0)	0.071
-Recurrent	7 (15.2)	7 (35.0)	
Leakage type, No. (%)			
-Smoke stack	8 (17.4)	3 (15.0)	0.652
-Ink blot	27 (58.7)	14 (70.0)	
-Diffuse	11 (23.9)	3 (15.0)	
GLD mean±SD (*μm*)	1324.50±1510.09	1957.70±1122.72	0.980
PDT spot size, mean±SD (*μm*)	2438.04±1649.09	3155.40±1450.59	0.980
Initial BCVA			
-LogMAR, mean±SD	0.40±0.37	0.40±0.41	0.908
Initial CRT, mean±SD (μm)	366.57±127.69	352.90 ±107.10	0.677

*Abbreviations*: *BCVA* best corrected visual acuity; *BMI* body mass index, *CRT* central retinal thickness; *GLD* greatest linear diameter; *LogMAR* logarithm of the minimum angle of resolution, *PDT* photodynamic therapy, *SD* standard deviation

### Changes in BCVA

The half-dose PDT group showed statistically significant BCVA improvement at 1, 3, 6 and 12 months after PDT treatment when compared with the baseline (Table 2). In the one-third-dose PDT group, BCVA showed statistically significant improvement at 6 and 12 months after PDT treatment, when compared with the baseline.

**Table 2:** The best corrected visual acuity (BCVA) results from patients with chronic or recurrent central serous chorioretinopathy before and after different doses of photodynamic therapy

LogMAR BCVA	Half-dose^(1)^ (mean±SD)	One-third-dose^(2)^ (mean±SD)	*P*-value^(1)–(2)^
At baseline ^a^	0.41±0.37	0.40±0.41	0.893
At 1 month ^b^	0.32±0.34	0.36±0.41	0.690
At 3 months ^c^	0.29±0.33	0.35±0.41	0.623
At 6 months ^d^	0.24±0.32	0.31±0.36	0.541
At 12 months ^e^	0.24±0.34	0.30±0.43	0.526
P-value	*a-b; p = 0.002, a-c, p < 0.001* *a-d, p < 0.001, a-e, p < 0.001*	*a-b; p =0.419, a-c, p= 0.216* *a-d; p =0.038, a-e; p =0.028*	

^a,b,c,d^: Linear fixed- and random-effects models

^(1)–(2)^: Linear fixed- and random-effects models

*Abbreviations*: *SD* standard deviation

When the two groups were compared, the half-dose PDT group did not show a statistically significant difference in BCVA (Table 2), or in BCVA improvement (Table 3) at 1, 3, 6 and 12 months; relative to the one-third-dose PDT group.

**Table 3:** Best corrected visual acuity (BCVA) improvement in patients with chronic or recurrent central serous chorioretinopathy after different doses of photodynamic therapy

BCVA improvement (LogMAR)	Half dose (mean±SD)	One-third dose (mean±SD)	*P*-value ^a^
*VA 1 month - VA Initial*	−0.09±0.26	− 0.04±0.13	0.317
*VA 3 month - VA Initial*	−0.11±0.27	− 0.05±0.15	0.244
*VA 6 month - VA Initial*	−0.16±0.30	− 0.09±0.14	0.161
*VA 12 month - VA Initial*	−0.17±0.29	− 0.10±0.17	0.148

^a^ Linear fixed- and random-effects models

*Abbreviations*: *BCVA* best corrected visual acuity, *LogMAR* logarithm of the minimum angle of resolution;

*SD* standard deviation

### Changes in CRT

Both groups showed statistically significant reductions in CRT, compared with the baseline, at 1, 3, 6 and 12 months after PDT treatment (Table 4). In contrast, the half-dose PDT group did not show statistically significantly differences in CRT or CRT reduction at 1, 3, 6 and 12 months, compared with the one-third-dose PDT group at these times (Tables 4 and 5).

**Table 4:** The central retinal thickness (CRT) in patients with chronic or recurrent central serous chorioretinopathy before and after different doses of photodynamic therapy

CRT (μm)	Half-dose^(1)^ (mean±SD)	One-third-dose^(2)^ (mean±SD)	*P*-value^(1)–(2)^
At baseline ^a^	366.57±127.69	352.90±107.10	0.501
At 1 month ^b^	237.48±57.92	221.55±38.15	0.434
At 3 months ^c^	244.39±84.14	222.11±31.76	0.290
At 6 months ^d^	227.87±48.91	228.85±55.54	0.949
At 12 months ^e^	227.46±52.20	227.35±34.78	0.758
P-value	*a-b, a-c, a-d, a-e; p < 0.001*	*a-b, a-c, a-d, a-e; p < 0.001*	

^a,b,c,d^: Linear fixed- and random-effects models

^(1)–(2)^: Linear fixed- and random-effects models

*Abbreviations*: *CRT* central retinal thickness, *SD* standard deviation

**Table 5:** Central retinal thickness (CRT) reduction in patients with chronic or recurrent central serous chorioretinopathy after different doses of photodynamic therapy

Mean CRT reduction (μm)	Half dose (mean±SD)	One-third dose (mean±SD)	*P*-value ^a^
*CST Initial - CST 1 month*	129.09±119.96	131.35±107.81	0.926
*CST Initial - CST 3 months*	122.17±137.57	132.63±107.63	0.738
*CST Initial – CST 6 months*	139.31±118.72	124.05±112.48	0.536
*CST Initial - CST 12 months*	139.11±112.16	125.55±110.07	0.933

^a^ Linear fixed- and random-effects models

*Abbreviations*: *CRT* central retinal thickness, *SD* standard deviation

### The accumulated number of eyes with complete SRF reabsorption after a single PDT treatment

In both groups, all eyes had evidence of SRF reabsorption within 12 months after a single PDT treatment (Table 6).

**Table 6:** The cumulative number of eyes with complete subretinal fluid reabsorption after a single photodynamic therapy treatment (PDT)

Outcome	Half-dose PDT group *N* (%)	One-third-dose PDT group *N* (%)	*P*-value
At 1 month			
Completely reabsorbed (*n*, %)	33 (72%)	12 (60%)	0.347
At 3 months			
Completely reabsorbed (*n*, %)	39 (85%)	17 (85%)	0.982
At 6 months			
Completely reabsorbed (*n*, %)	44 (96%)	19 (95%)	0.907
At 12 months			
Completely reabsorbed (*n*, %)	46 (100%)	20 (100%)	

### The cumulative number of eyes with recurrence of SRF after a single PDT treatment

In the half-dose PDT group, the accumulated numbers of eyes with recurrence of SRF after achieving a dry macula were 2 (4.3%), 4(8.7%) and 7(15.2%) within 3, 6 and 12 months after a single PDT treatment (Table 7). Two eyes had recurrent SRF within 3 months and were successfully treated with a repeated half-dose PDT. Two eyes had recurrence within 6 months; one was successfully treated with focal laser, while the other spontaneously resolved with observation.

**Table 7:** The cumulative numbers of eye with recurrence of subretinal fluid (SRF) after a single photodynamic therapy treatment (PDT)

Recurrent SRF	One-third-dose PDT group *N* (%)	Half-dose PDT group *N* (%)	*P*-value
Within 3 months	1 (5%)	2 (4.3%)	0.907
Within 6 months	5 (25%)	4 (8.7%)	0.076
Within 12 months	8 (40%)	7 (15.2%)	0.027*

In the one-third-dose PDT group, the cumulative numbers of eyes with recurrent SRF after a dry macula, were 1(5%), 5(25%) and 8(40%) within 3, 6 and 12 months after PDT treatment. One eye had a recurrence within 3 months and was successfully treated with a repeated one-third-dose PDT. Four eyes had SRF recurrence within 6 months. In 3 cases, these were successfully treated with a repeated one-third-dose PDT. The other case spontaneously resolved after observation. In both groups, three eyes had recurrence of SRF at their 12-month follow up.

The one-third-dose PDT group showed a significantly, higher recurrence rate than that of the half-dose PDT group at 12 months (*P* = 0.027) (Table 7).

#### The comparison of demographic data of the patients with recurrence of subretinal fluid, after photodynamic therapy, between the two treatment groups

The comparison of demographic data of the patients with recurrence of subretinal fluid after photodynamic therapy, between the two treatment groups is summarized in Table 8. There were no statistically significant differences in age, gender, laterality, body mass index, duration of symptoms, type of CSC, leakage type on FA, spot size of PDT, initial BCVA and initial CRT.

**Table 8:** Comparison of demographic data for patients with recurrence of subretinal fluid, after photodynamic therapy, between the two treatment groups

Demographic (*N*=15)	Half-dose PDT group (*n* = 7eyes/ 5patients)	One-third-dose PDT group (*n* = 8eyes/7 patients)	*P*-Value
Age mean±sd (years)	52.00±6.44	48.14±9.99	0.469
Sex,No.(%)			
Male	5 (100)	7 (100)	–
Female	0 (0.0)	0 (0.0)	
Study eye, No. (%)			
RE	4 (57.1)	3 (37.5)	0.619
LE	3 (42.9)	5 (62.5)	
BMI,mean±sd,kg/m^2^	23.81±6.85	24.99±3.30	0.697
duration of symptom, mean±sd, (months)	15.00±11.59	16.25±8.69	0.815
Group Type			
recurrent	0 (0.0)	3 (37.5)	0.200
chronic recurrent	7 (100)	5 (62.5)	
Leakage type, No. (%)			
Smoke stack	0 (0.0)	1 (12.5)	0.348
Ink blot	2 (28.6)	4 (50.0)	
Diffuse	5 (71.4)	3 (37.5)	
GLP (mean±sd)	2187.14±1901.31	2339.00±1457.98	0.864
PDT spot size, (mean±sd)	3514.29±2132.62	3638.38±2080.62	0.911
Initial BCVA			
LogMAR, (mean±sd)	0.62±0.38	0.30±0.33	0.099
Initial CRT, (mean±sd)	321.00±135.67	288.25±60.02	0.546

*Abbreviations*: *BCVA* best corrected visual acuity; *BMI* body mass index, *CRT* central retinal thickness, *GLD* greatest linear diameter; *LogMAR*, logarithm of the minimum angle of resolution, *PDT* photodynamic therapy, *SD* standard deviation

After recurrence SRF, with a single PDT, 6/7 eyes in the half-dose PDT groups and 7/8 eyes in the one-third-dose PDT group were reperformed FA. In the half-dose PDT group, the 5/6 eyes and 1/6 eyes consisted of leaks in area having been previously treated, and de-novo leakage, respectively. In the one-third-dose PDT group, the 6/7 eyes and 1/7 eyes consisted of leaks in area having been previously treated and de-novo leakage, respectively.

#### Safety

There were no patients in either group who had an allergic reaction to verteporfin, and there were no ocular adverse events; including any development of secondary CNV, during the follow-up periods.

## Discussion

CSC is characterized by an accumulation of SRF in the macula, with or without serous detachment of the RPE, caused by choroidal vascular hyperpermeability [1, 2]. PDT is applied for treatment of CSC because it acts to reconstruct the choroidal vasculature and reduce vascular hyperpermeability [8–13]. Recently, the PDT protocol has been modified to reduce the rate of complications, by reducing the dosage of verteporfin or reducing the fluence [17–23]. However, to-date, the optimal PDT protocol for maintaining efficacy, and reducing adverse events remains unclear. This retrospective study compared the efficacy of one-third-dose and half-dose PDT for chronic, or recurrent CSC.

The reduction of CRT in this study was similar to that in previous studies [17, 23–26]. The previous studies using half-dose PDT [17, 23, 26] showed that the CRT reduction was significant from 1 month, until 12-months of follow-up. Similarly, in previous studies using one-third-dose PDT, Zhoa et al. [24, 25] showed that CRT was significantly reduced from 1 month to the 12-months of follow-up. In our study, both half-dose and one-third-dose PDT were effective in reducing CRT in both chronic and recurrent CSC from, 1 month until 12 months after treatment.

Uetani et al. [27] reported that the SRF reabsorption rates were 70 and 33% at 1 month after treatment with half-dose and one-third-dose PDT, respectively, in patients with chronic CSC. Similarly, Zhoa et al. [25] reported that the SRF reabsorption rates were 92.9 and 73.8% at 6 months after treatment with a single half-dose and one-third-dose PDT, respectively, in chronic CSC. In our study, the same tendency was observed. The half-dose PDT group showed a higher percentage of eyes with SRF reabsorption than that of the one-third-dose PDT group (72% versus 60%; although the difference was not significant), at 1-month post-treatment. Because of the small number of eyes in this study, the speed with which SRF reabsorption occurred after PDT in the two groups requires further study.

The BCVA improvements observed in this study were similar to that in previous studies [17, 23–26]. Previous studies using half-dose PDT [17, 23, 28] showed that BCVA was significantly improved at 12 months after PDT. Similarly, a study by Zhoa et al. [25]; using one-third-dose PDT, showed significant improvement in BCVA at the 12-month follow-up period. In our study, both half-dose and one-third-dose PDT were effective in improving BCVA in chronic, or recurrent CSC at 12 months. However, the half-dose PDT group showed faster BCVA improvement than that of the one-third-dose PDT group. This result may be due to the higher percentage of early SRF reabsorption in the half-dose PDT group.

Many previous studies [17, 23] have reported 1-year recurrence rates of SRF in half-dose PDT of approximately 8.3–13.2%. In our study, the rates were 15.2% in the half-dose PDT group and 40% in the one-third-dose PDT group. We postulate that the higher recurrence rate in the one-third-dose PDT group may have been caused by the lower effect on choroidal remodeling and choroidal hyperpermeability with reduced doses of verteporfin. However, the changes in choroidal thickness after different doses of PDT should be demonstrated in further studies.

This study showed that one-third-dose PDT was effective in the treatment of chronic or recurrent CSC. However, our study also showed that the recurrence rate with the one-third-dose was significantly higher than that in the half-dose PDT within 1 year after treatment. Therefore, in cases of chronic or recurrent CSC, half-dose PDT should be recommended, more so than the one-third dose regimen. The limitations of this study are its retrospective nature, having a small sample size in the one-third-dose PDT group, and the lack of comparison of choroidal thicknesses between groups.

## Conclusion

In conclusion, these 1-year results show one-third-dose PDT was effective in term of BCVA and CRT improvement when compared with half-dose PDT. However, the one-third-dose PDT had a higher recurrent rate of disease.

## Data Availability

The datasets used and/or analyzed during the current study are available from the corresponding author upon reasonable request.

## Abbreviations

PDT: Photodynamic therapy
CSC: Central serous chorioretinopathy
BCVA: Best-corrected visual acuity
CRT: Central retinal thickness
SRF: Subretinal fluid
RPE: Retinal pigment epithelium
CNV: Choroidal neovascularization
SD-OCT: Spectral-domain optical coherence tomography
FA: Fluorescein angiography
VEGF: Vascular endothelial growth factor
PCV: Polypoidal choroidal vasculopathy
ICGA: Indocyanine green angiography
